# MicroRNAs in urine are not biomarkers of multiple myeloma

**DOI:** 10.1186/s12952-015-0035-7

**Published:** 2015-09-23

**Authors:** Lenka Sedlaříková, Lenka Bešše, Soňa Novosadová, Veronika Kubaczková, Lenka Radová, Michal Staník, Marta Krejčí, Roman Hájek, Sabina Ševčíková

**Affiliations:** Babak Myeloma Group, Department of Pathological Physiology, Faculty of Medicine, Masaryk University, Brno, Czech Republic; Department of Clinical Hematology, University Hospital Brno, Brno, Czech Republic; Department of Hematology and Oncology, Kantonsspital St. Gallen, St. Gallen, Switzerland; Central European Institute of Technology, Masaryk University, Brno, Czech Republic; Department of urologic oncology, Masaryk Memorial Cancer Institute, Brno, Czech Republic; Department of Internal Medicine - Hematooncology, University Hospital Brno, Brno, Czech Republic; Department of Hematooncology, Faculty of Medicine University of Ostrava and University Hospital Ostrava, Ostrava, Czech Republic

## Abstract

**Background:**

In this study, we aimed to identify microRNA from urine of multiple myeloma patients that could serve as a biomarker for the disease.

**Results:**

Analysis of urine samples was performed using Serum/Plasma Focus PCR MicroRNA Panel (Exiqon) and verified using individual TaqMan miRNA assays for qPCR. We found 20 deregulated microRNA (*p* < 0.05); for further validation, we chose 8 of them. Nevertheless, only differences in expression levels of miR-22-3p remained close to statistical significance.

**Conclusions:**

Our preliminary results did not confirm urine microRNA as a potential biomarker for multiple myeloma.

## Background

Multiple myeloma (MM) is a malignancy of plasma cells (PCs) that manifests also by renal insufficiency [1]. MicroRNAs (miRNAs) are small non-coding single stranded RNAs and important regulators of gene expression involved in MM pathogenesis [2]. Currently, research attention is focused on circulating miRNAs which can be detected in various body fluids, also in urine. Circulating miRNAs are highly stable and have the potential to become easily available minimally invasive biomarkers of the disease [3]. For MM, they would represent a new more convenient approach since painful and invasive bone marrow (BM) biopsy is used for disease diagnostics and monitoring. We hypothesized that miRNAs identified in urine of MM patients could become a completely non-invasive biomarker potentially distinguishing MM patients from healthy donors (HD).

## Results and discussion

**Fig. 1:**
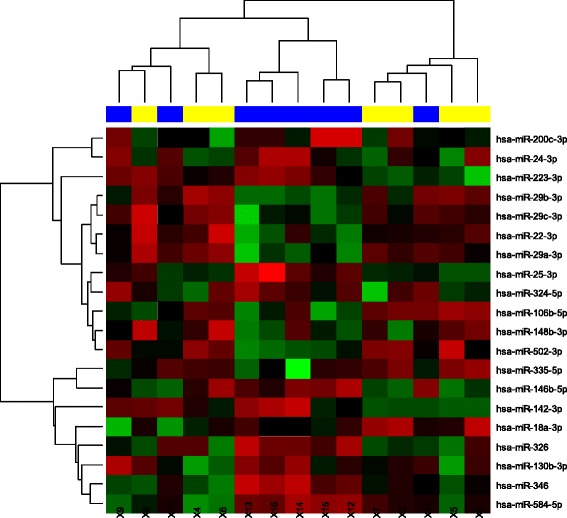
Hierarchical clustering of differentially expressed miRNAs from urine between MM patients (yellow) and HD (blue) using heatmaps. Different expression is expressed by range of red (overexpression) vs green (low expression) colors

**Fig. 2:**
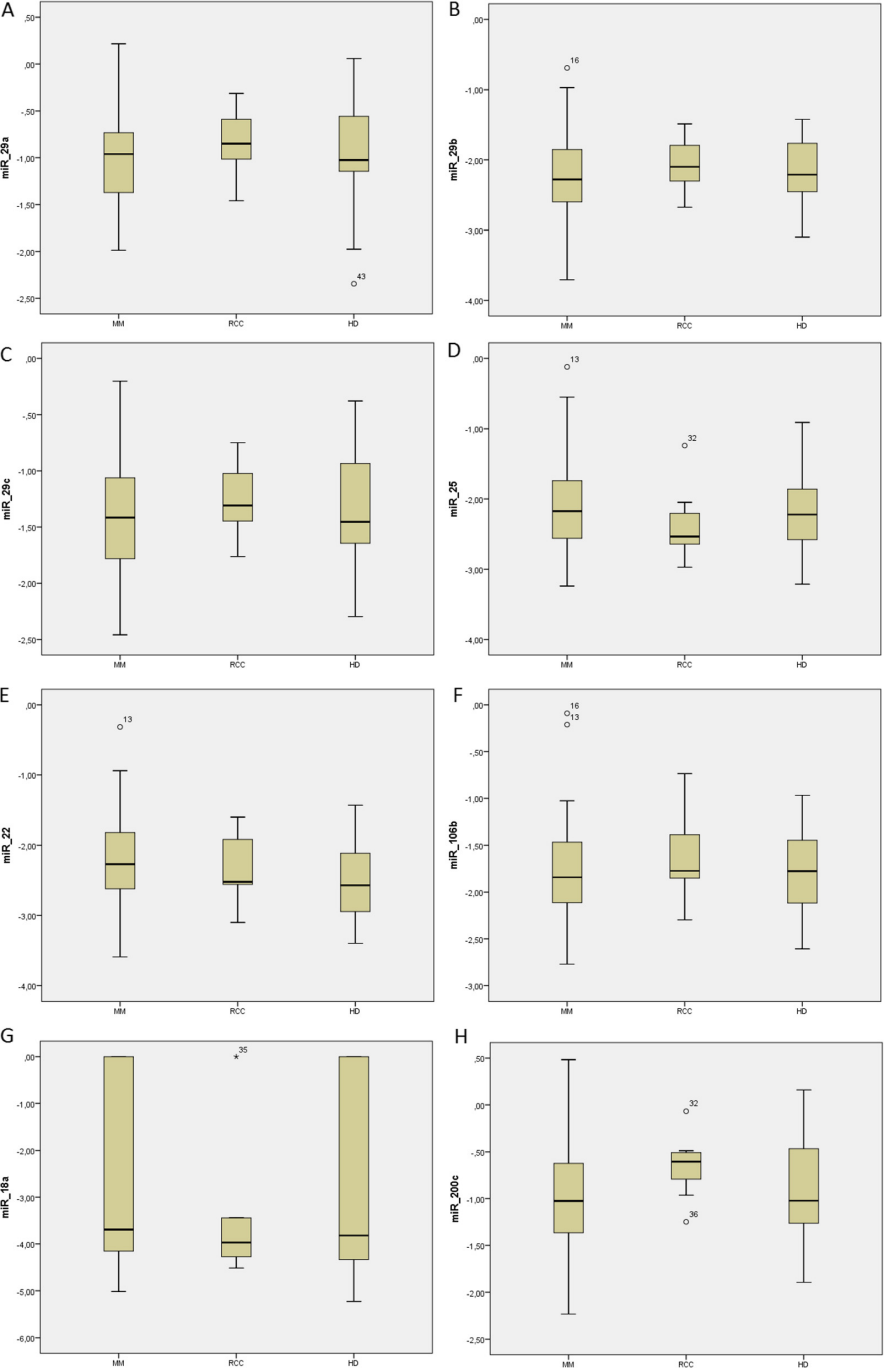
Comparison of expression levels of validated miRNAs defined as logarithmic values of 2^-ΔCt^ normalized to cel-miR-39 expression levels for MM, RCC patients and HD. **a** – miR-29a-3p; **b** – miR-29b-3p; **c** – miR-29c-3p; **d** miR-25-3p; **e** – miR-22-3p; **f** – miR-106b-5p; **g** – miR-18a-3p; **h** – miR-200c-3p

Screening of miRNA in urine revealed 20 deregulated miRNAs (*p* < 0.05 for all miRNAs) between MM and HD (Fig. 1). For further validation, we chose 8 most deregulated miRNAs between MM and HD: miR-200c-3p, miR-29b-3p, miR-29c-3p, miR-22-3p, miR-29a-3p, miR-25-3p, miR-106b-5p, miR-18a-3p (Table 1). Expression levels of these miRNAs were further verified on a larger cohort of MM patients, RCC patients and HD. Results from validation revealed that expression levels of chosen miRNAs from urine of MM patients are not statistically different (*p* = 0.05) from HD and RCC patients (Fig. 2). Results from validation did not confirm statistical significance from the screening. Only differences in expression levels of miR-22-3p between MM patients and HD in urine (*p* = 0.090) remained close to statistical significance (Table 2).

**Table 1:** *P* values and sample size estimation for differentially expressed miRNAs in the screening phase

miRNA	*P* value	*adj.P* value	Sample size
hsa-miR-29b-3p	0.0043	0.2074	25.14
hsa-miR-29a-3p	0.0060	0.2074	81.74
hsa-miR-22-3p	0.0062	0.2074	58.68
hsa-miR-29c-3p	0.0111	0.2074	36.19
hsa-miR-25-3p	0.0176	0.2074	55.67
hsa-miR-106b-5p	0.0195	0.2074	32.32
hsa-miR-18a-3p	0.0303	0.2784	125.39
hsa-miR-200c-3p	0.0426	0.3121	29.50

**Table 2:** Validated microRNAs

miRNA	HD	MM	RCC	*P* value		
	mean	mean	mean			
	(25–75)	(25–75)	(25–75)	HD vs. MM	RCC vs. MM	HD vs. RCC vs. MM
	Std. deviation	Std. deviation	Std. deviation			
miR-29a	−1.007	−0.984	−0.833	0.863	0.466	0.739
	(−1.145) - (−0.556)	(−1.384) - (−0.715)	(−1.172) - (−0.475)			
	0.559	0.506	0.393			
miR-29b	−2.204	−2.248	−2.064	0.570	0.394	0.581
	(−2.458) - (−1.751)	(−2.598) - (−1.848)	(−2.352) - (−1.493)			
	0.446	0.580	0.438			
miR-29c	−1.386	−1.426	−1.252	0.663	0.329	0.565
	(−1.656) - (−0.933)	(−1.796) - (−1.040)	(−1.570) - (−0.856)			
	0.482	0.518	0.361			
miR-25	−2.224	−2.106	−2.347	0.692	0.329	0.608
	(−2.593) - (−1.848)	(−2.595) - (−1.717)	(−2.742) - (−2.049)			
	0.598	0.634	0.567			
miR-22	−2.532	−2.247	−2.310	0.090	0.790	0.225
	(−2.954) - (−2.084)	(−2.744) - (−1.782)	(−2.566) - (−1.676)			
	0.538	0.689	0.536			
miR-106b	−1.788	−1.765	−1.612	0.958	0.496	0.776
	(−2.120) - (−1.419)	(−2.138) - (−1.423)	(−1.901) - (−1.102)			
	0.460	0.555	0.524			
miR-18a	−2.736	−2.824	−3.416	0.763	0.528	0.802
	(−4.359) - (0.000)	(−4.171) - (0.000)	(−4.303) - (−3.437)			
	2.101	1.874	1.563			
miR-200c	−0.909	−0.990	−0.645	0.543	0.088	0.237
	(−1.273) - (−0.449)	(−1.405) - (−0.588)	(−0.963) - (−0.487)			
	0.610	0.595	0.374			

Expression levels defined as logarithmic values of 2^-ΔCt^ normalized to cel-miR-39 expression levels as mean value and interquantile range with standard deviation. Nonparametric Kruskal-Wallis or Mann–Whitney U test was used to compare the values

Nowadays, there is increasing evidence of miRNAs importance in MM pathogenesis. MiRNA expression profiles could be useful for MM stratification, prognostic estimation, prediction of therapeutic effectiveness or disease relapse [2, 4]. Moreover, miRNAs could potentially provide deeper insight into molecular nature of the disease and help to develop miRNAs-based therapeutic agents subsequently improving patients’ outcome [5]. It has been shown that circulating miRNAs found in various body fluids may serve as a new class of powerful and minimally invasive biomarkers for MM. Although several studies describing circulating miRNAs in peripheral blood of MM patients were published [6–8], no study focused on miRNA expression profiles in urine of MM patients has been published so far.

As one of the typical clinical manifestations of MM is renal insufficiency, included as one of the ‘CRAB’ criteria for organ damage in MM [1], we hypothesized that miRNAs found in urine of MM patients could serve as diagnostic biomarkers of the disease. Twenty differentially expressed miRNAs between MM and HD urine samples were identified using Serum/Plasma Focus PCR MicroRNA Panel; out of them, eight miRNAs were chosen for further analysis. Unfortunately, none of them was assessed as significantly deregulated in a larger cohort of MM, RCC patients and HD using individual TaqMan miRNA assays. In our study, we included not only MM patients and HD, but also RCC patients to exclude miRNA that are related to kidney damage.

As no differences in miRNA expression were found in the validation phase of the study, we considered several reasons for this outcome. First reason is possible analytical difference between the screening and validation phase of our study. We believe that using different detection approaches (Serum/Plasma Focus PCR MicroRNA Panel versus individual TaqMan miRNA assays) was not the reason for discordance between the screening and validation phase since they are both reliable methods verified by many researchers [9, 10]. Second reason is differences in sample collection and processing. We believe that the discordance was not caused by disunity of samples as they were all collected and processed in the same manner. Third possibility is using cel-miR-39 as a spike-in control for normalization. This approach is now considered a suitable approach in relative quantification [11], and it was applied in both phases of the study. On the other hand, a small cohort of patients used in the screening phase may be considered a major limitation as it may not be powerful enough for identification of a biomarker. Although the cohort seems to be small, it is a standard way of identifying possible differences of miRNA expression [8, 10]. While it is possible that we could have missed some significantly differently expressed miRNAs, we found twenty miRNAs to be deregulated (*p* < 0.05 for all miRNAs). In the design of our study, the screening phase was not a test cohort, but a way of finding significant miRNAs which should be studied further; we believe that the screening phase fulfilled this purpose.

Another possibility to be considered is the existence of previously published work that identified urine miRNAs as markers of various diseases – such work has been done, for example in urologic cancers (reviewed in [12]). Urinary miRNAs are easily accessible and quantifiable and thus have a great potential to become biomarkers in oncology and nephrology [12]. It has been previously published that urinary miRNAs may be used for diagnosis and monitoring of urothelial carcinoma (UC). Deregulated levels of miR-126, miR-96, miR-200 family and miR-183 family were repeatedly observed in UC patients. Also, it was observed that increased expression levels of urinary miR-15a could serve as a biomarker for benign/malignant RCC differentiation [12].

Moreover, some of studied urinary miRNAs were previously described in MM PCs [4, 13, 14]. Members of the miR-29 family (miR-29a-3p, miR-29b-3p and miR-29c-3p) were found in MM PCs with decreased expression levels; also, presence of circulating miR-29a was detected in serum of MM patients [13, 15]. Apart from hematological malignancies, increased expression levels of these miRNAs were found in RCC and decreased expression levels in diabetic nephropathy (DN) [16].

Overexpression of cluster miR-106b-25 seems to be contributing to transformation of monoclonal gammopathy of undetermined significance (MGUS) patients to MM as its expression levels are increased in both, MGUS and MM PCs compared to HD [2]. Increased expression levels of miR-106b occur in RCC cells as well. It was suggested that expression levels of this miRNA could become a predictive biomarker for metastasis formation after surgical removal of kidneys [14]. On the other hand, miR-22 expression levels were found to be decreased in MM cell lines and were associated with 17p deletion [4].

MiR-18a is member of the miR-17-92 cluster which is present in PCs of MM patients with higher expression levels. MiRNAs originating from this cluster of genes promote leukemogenesis. The miR-17-92 cluster is activated by Myc and subsequently downregulates proapoptotic protein Bim thus promoting MM cell proliferation and inhibition of cell apoptosis. Moreover, the miR-17-92 cluster is linked to MM progression and poor prognosis [17]. On contrary, decreased expression levels of miR-18a was detected in bladder cancer [14, 18]. MiR-200c has not been found in MM PCs so far. However, its decreased expression levels were shown in RCC and DN [19].

## Conclusions

In conclusion, we have identified several miRNAs in urine of MM patients that were previously described to be involved in MM pathogenesis or kidney-associated diseases but are not disease-specific. Thus, we could not confirm our hypothesis that there is a set of circulating urinary miRNA that could serve as a non-invasive marker of MM.

## Methods

In total, 85 urine samples were included in the study (Table 3). Samples were collected as 8 mL of urine stabilized by 0.149 g of EDTA. MiRNAs from 1 mL of urine were isolated using Urine MicroRNA Purification Kit (Norgen Biotek, Canada) according to manufacturer’s recommendations and quantified using Nanodrop-ND1000 spectrophotometer. 40 ng of isolated miRNA was reverse transcribed by Universal cDNA Synthesis Kit (Exiqon, Denmark). Analysis of potentially biologically significant miRNAs in urine was performed using Serum/Plasma Focus PCR MicroRNA Panel (Exiqon, Denmark) determining expression levels of 179 miRNAs of 7 urine samples of newly diagnosed MM patients and 8 HD. Normalized expression data from the screening phase of the study were statistically analyzed by freeware R/Bioconductor and its additional packages. LIMMA approach was used to identify differentially expressed miRNAs with Benjamini-Hochberg adjustment of P values. To clarify similarity of samples, hierarchical clustering was applied. Individual TaqMan miRNAs assays for 8 differentially expressed miRNAs (hsa-miR-200c-3p, hsa-miR-29b-3p, hsa-miR-29c-3p, hsa-miR-22-3p, hsa-miR-29a-3p, hsa-miR-25-3p, hsa-miR-106b-5p, hsa-miR-18a-3p, Life Technologies, USA) were used for qPCR on 7500 Real-Time PCR System. qPCR and reverse transcription using TaqMan MicroRNA Reverse Transcription Kit (Life Technologies, USA) was performed following manufacturer’s recommendations. Results were obtained by relative quantification using spike-in controls cel-miR-39 in 49 newly diagnosed MM, 20 HD and 7 patients with renal cell carcinoma (RCC) in order to distinguish miRNAs associated with MM. Analytical performance of the study was assessed by intraplate and interplate controls. Standard descriptive statistics were applied in the analysis; median supplemented by interquartile range for continuous variables. Statistical significance of differences in continuous variables among groups of patients was analyzed using nonparametric Kruskal-Wallis or Mann–Whitney U test. Statistical analysis of data from validation phase of the study was performed using IBM SPSS Statistics, v. 20. The study was approved by the Ethics committee of the University Hospital Brno. All patients were included into the study only after they signed the informed consent form.

**Table 3:** Patients’ characteristics

	HD	RCC	MM
No. of patients/donors	22	7	56
Gender: males-females	9-13	6-1	28-28
Age median (min-max) [years]	57 (50–90)	61 (27–83)	69 (49–89)
ISS stage: I-II-III	ND	ND	16-12-19
D-S stage: I-II-III	ND	ND	3-7-40
D-S substage: A-B	ND	ND	39-11
Ig isotype: IgG-IgA-FLC	ND	ND	34-11-10
Light chains: kappa-lambda	ND	ND	40-15
Amount of M-Ig/FLC g/l median (min-max)	ND	ND	25,45 (0,17-65,4)
PCs infiltration in BM	ND	ND	13,1 (0,14-87,4)
No treatment	ND	ND	56 (100 %)

ND – not done, ISS stage – International Staging System stage, D-S stage – Durie-Salmon stage, M-Ig – monoclonal immunoglobulin, FLC – Free Light Chains

## Abbreviations

BM: bone marrow
DN: diabetic nephropathy
HD: healthy donors
miRNAs: microRNAs
MGUS: monoclonal gammopathy of undetermined significance
MM: multiple myeloma
PCs: plasma cells
RCC: renal cell carcinoma
UC: urothelial carcinoma
